# Transposons disrupt genomic stability and trigger cancers

**DOI:** 10.3389/fcimb.2025.1704405

**Published:** 2025-12-12

**Authors:** Weixia Dong, Menghui Li, Ping Li, Xiaoli Hou, Fei He, Shaoping Ji

**Affiliations:** 1 Henan Provincial Research Center of Engineering Technology for Nuclear Protein Medical Detection, Zhengzhou Health College, Zhengzhou, Henan, China; 2 Translational Medicine Center, Huaihe Hospital Affiliated to Henan University, Henan, China; 3 Department of Biochemistry and Molecular Biology, Medical School, Henan University, Kaifeng, Henan, China

**Keywords:** transposons, genomic stability, regulation, insert, cancer

## Abstract

Transposons (TEs) are genetic elements that can change their positions within genome. They cause transcriptional activation or repression by inserting into regulatory elements of related genes or by modulating epigenetic modifications on regulatory elements. In addition, some transposons encode and express peptides or proteins that affect or disrupt the cellular biological functions. Recent studies have demonstrated that while host cells effectively control most transposons, certain harmful insertions can disrupt normal gene expression processes, potentially leading to diseases. In this mini-review, we discuss recent advances in understanding how transposons contribute to genomic instability. We hope to provide researchers and clinical practitioners with new insights into transposon biology and its potential implications for disease pathogenesis.

## 1 Introduction

Transposons can be classified into two classes. The class I (retro-transposons), is the largest category of mobile genetic elements, utilizing a ‘copy-and-paste’ mechanism mediated through RNA intermediates. These elements are first transcribed into RNA, then reverse-transcribed into cDNA copies that integrate at new genomic loci (Bourque et al., 2018). As a result, they generate many repetitive sequences in higher eukaryotic organisms (De Bustos et al., 2016), which can disturb gene expression. Moreover, reverse transcription transposons are further sub-classified into long terminal repeat (LTR) reverse transcription transposons and non-LTR reverse transcription transposons, both include long and short interspersed nuclear elements (LINEs and SINs). Class II transposons move in a “cut and paste” pattern, jumping from one genomic location to another by a transposase coded by itself (Redd et al., 2023). The disruption of genome by Class II movement is caused by imperfect gap repair, leading to mutation and new insertion mutation in genome (Kawakami et al., 2017).

As a mobile genetic element, transposons insert along the genome, impacting genomic stability and gene expression by interfering with regulatory elements and/or disrupting coding sequences. They may cause transcriptional activation or repression if inserted into the regulatory elements of related genes (Fueyo et al., 2022). For example, the insertion of ERV repeat sequences upstream of the *AMY1* gene can lead to the activation of a cryptic promoter and drive tissue-specific expression of the gene (Samuelson et al., 1990). Studies have shown that somatic retrotransposon integration occurs in about 1 in 2 cancers. LINE-1(L1)insertions are the most common type of somatic structural variation in esophageal adenocarcinomas and the second most common in head and neck as well as colorectal cancers. Abnormal L1 retro-transposition can lead to the deletion of tumor suppressor genes and the amplification of oncogenes (Rodriguez-Martin et al., 2020). This mobility contributes to various disorders, including cancer (Fukuda, 2024). Lee E. et al. performed single-nucleotide resolution analyses of tumor genomes from five cancer types and found that somatic retrotransposition of L1 elements predominantly occurs in epithelial tumors. These insertions frequently target genes that are commonly mutated in cancer, disrupt the expression of target genes, and tend to localize within cancer-specific hypomethylated genomic regions. These findings suggest that dysregulated transposon activity may act as a driving force in tumorigenesis (Lee et al., 2012). Alternatively, they can interfere with gene expression via epigenetic modification (McCue et al., 2012). Transposons can escape epigenetic silencing, and hypomethylation combined with dysregulated chromatin modifications of L1 retrotransposons can promote their insertion into novel genomic sites, a phenomenon observed in pancreatic ductal adenocarcinoma and esophageal squamous cell carcinoma (Gebrie, 2023).

Some transposons integrate into the genome and become immobile, eventually becoming part of the human genome. However, they may still affect gene expression by acting as regulatory elements or being transcribed through different mechanisms (Chesnokova et al., 2022). Transposons direct participation in gene regulation via transposon-derived *cis*-regulatory regions and transposon-derived regulatory RNA sequences in the human genome. Transposons may carry intrinsic regulatory sequences, such as promoters, enhancers, splice sites, and terminators, and different types of transposon have distinct features; for example, LTRs and LINEs carry Pol II promoters, which can influence gene expression (Ali et al., 2021). Some transposons, such as L1 elements, can also produce antisense transcripts that extend into neighboring genes, forming chimeric RNAs and potentially interfering with normal gene expression (Speek, 2001). HERV-H retroelements can function as enhancers, driving the expression of pluripotency-associated long noncoding RNAs in human embryonic stem cells. Disruption of HERV-H or its derived transcripts leads to changes in cell morphology and a reduction in pluripotency, indicating a critical role in maintaining stem cell identity (Ali et al., 2021). Thus, both mobile and fixed transposons can alter genomic stability and gene expression, though most insertions are usually harmless to gene function.

## 2 Transposons insert themselves into genomes in various patterns

Given the significant contribution of transposable elements to human genetic variation and disease, it is essential to understand the insertion patterns of transposon subfamilies that remain active and mobile in contemporary human populations. In the human genome, three non-LTR retrotransposon families, Alu, L1, and SVA, remain transcriptionally active and continue to drive new mobile element insertions (MEIs) (Devine, 2023). L1 elements integrate into the genome via target-primed reverse transcription (TPRT): in the cytoplasm, L1 RNA forms a ribonucleoprotein complex (RNP) with ORF1p and ORF2p, which subsequently enters the nucleus. The endonuclease domain of ORF2p cleaves the DNA at specific target sites, providing a primer for reverse transcription. Insertions are typically accompanied by target site duplications (TSDs), a 3′ polyA tail, 5′ truncations, and occasionally, internal 5′ inversions generated by the TPRT variant known as “twin priming” (Nummi et al., 2025). Non-autonomous elements like Alu and SVA rely on L1 enzymatic activity for mobilization (Ostertag et al., 2003). LTR retrotransposons (e.g.,HERVs) integrate via RNA intermediates and integrase-mediated mechanisms, generating long terminal repeats (LTRs) that can influence nearby promoters or enhancers (Bushman, 2003). DNA transposons utilize a cut-and-paste mechanism, directly excising and reinserting DNA, potentially causing small deletions or chromosomal rearrangements (Muñoz-López and García-Pérez, 2010). Different insertion patterns can disrupt genomic stability by activating oncogenes, inactivating tumor suppressor genes, altering regulatory elements, or producing non-coding RNAs and functional proteins, further contributing to cancer and other diseases. Understanding these insertion patterns aids in predicting pathogenic insertions, guiding genomic screening, and provides a theoretical foundation for developing transposon-targeted cancer therapies.

## 3 Host cells regulate the activity of inserted transposable elements

There are more than 4.5 million transposons insertions in the human genome. A large number of transposons are not only activated during early embryonic development, but also act as enhancers at the stage of tissue differentiation, helping to control the expression of nearby genes, for example by activating a class of genes known as KRAB zinc-finger proteins (KZFPs), which have emerged as evolutionary ‘control molecules’ to specifically repress the activities of transposons, which act in conjunction with the KZFP family that evolved during the same period. Transposon and the concurrently evolving KZFP family act together to form the gene regulatory network that shapes early human development through a combination of activation and repression (Pontis et al., 2022). In addition, host cells have evolved a series of mechanisms to inhibit transposon activity during evolution to maintain genome integrity and stability. The inserted transposons in genome are usually methylated, which established some methylated original sites, leading to extension of DNA methylation along genome (Estécio et al., 2012). Studies have shown that Scaffold Attachment Factor B (SAFB) binds to conserved adenosine-rich sequences within L1 retrotransposons. By preventing the exonization of integrated L1 sequences, SAFB maintains the integrity of host pre-mRNA splicing and consequently suppresses L1 retrotransposition activity, thereby ensuring genomic stability (Ilık İ et al., 2024). In germline cells, transposons transcriptions are usually suppressed via PIWI-interacting RNA pathway, or their RNAs are degraded after transcription (Czech et al., 2018). Mechanically, histone 3 lysine 9 and 36 methylation plays a vital role in repressing transposon transcription activation in genetic regulator mechanism (Enke et al., 2011; Lindehell et al., 2023). In a large-scale investigation, Walter Metal et al. shown that H3K9me3 and H3K27me3 primarily suppress transposon transcription/jumping and maintain genome stability in long-term state (Walter et al., 2016). Thus, it is well known that host cells maintain genomic homeostasis and stability against transposon threats via methylating transposon inserts, particularly in human germline cells (Sigurdsson et al., 2012). Zinc-finger MYM-type protein 2(ZMYM2), as a transcriptional repressor, plays a critical role in the embryonic development. It mediated germline gene silence via DNA methylation, including transposon-related elements. As a result, it extensively repressed transcription activation of genes during early embryo, where premature activation of some genes and/or transposons may impair early development (Graham-Paquin et al., 2023). Additionally, RNA N6-methyladenosine(m^6^A) reader YTH Domain-Containing 2 (YTHDC2)associated with Ten-Eleven Translocation Methyl-cytosine Dioxygenase 1 (TET1)to remove DNA 5-methylcytosine, leading transposon demethylation and transcription activation. Intriguingly, this interplay determines neural stem cell differentiation and specification (Sun et al., 2023), but whether its demethylation activity on transposons can lead to tumorigenesis remained unknown.

Human embryonic germline control of transposons and their co-option in development have been thoroughly reviewed (Oomen and Torres-Padilla, 2024), highlighting how these processes benefit embryo development through the controlled expression of certain transposons (DiRusso and Clark, 2023). Research showed transposon can express in early embryonic development, or in special cells such as germline cells, the placenta or cultured embryonic stem cells (Hackett et al., 2017). In these cells, there are more euchromatin observed than that in differentiated cells. It is well known that genes located in the euchromatin can be transcribed (Huisinga et al., 2006). Transposon expression may disturb stability of host cells, bringing disaster to host cells and leading to diseases (Gerdes et al., 2016). The piggyBac transposable element-derived 5 (PGBD5) gene is an active DNA transposase that induces site-specific DNA rearrangements, is defined as an oncogenic mutator, and plays a role in the majority of childhood solid tumors, including lethal rhabdoid tumors (Henssen et al., 2020). Some investigations indicated there is a high expression level of transposons in cancer cells (Grundy et al., 2022). Most transposons in genome are harmless either because they are silenced by host cells or they lost activity over evolutionary process (Oomen and Torres-Padilla, 2024). However, a global hypomethylation of genome was identified in cancer cells with high expression of transposon (Kanholm et al., 2023).

Although host cell can well govern transposon transcription/expression, transposons also spatiotemporally alter host gene expression in a random or accident manner (Zaratiegui, 2017; Yoth et al., 2022). Silenced transposons in genome can be activated with other virus infection, besides leading to immune response (Hale, 2022).To suppress transposon transcription, testis expressed 15(TEX15) plays a critical role in piRNA-directed transposon DNA methylation (Schöpp et al., 2020).

## 4 Transposons disrupt cellular functions by activating certain genes

**Figure 1:**
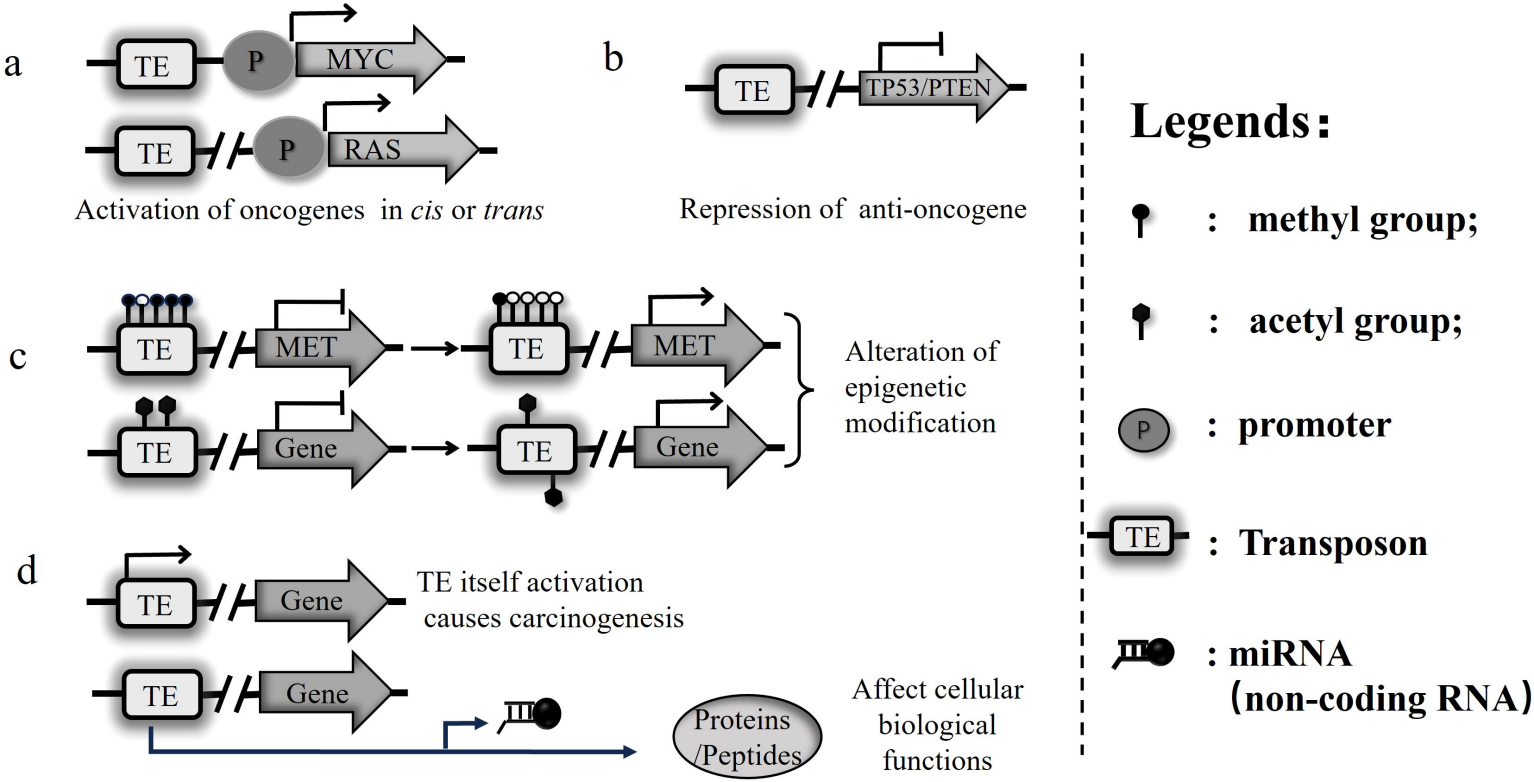
Transposons disrupt genomic stability through multiple mechanisms, driving tumorigenesis. **(a)** Transposons activate oncogenes via cis or trans mechanisms, promoting cancer development. **(b)** Insertion of transposons into tumor suppressor or other essential genes leads to their inactivation, facilitating tumorigenesis. **(c)** Transposon insertions within regulatory regions can alter transcriptional activation or repression, directly or through epigenetic modifications. **(d)** Beyond insertional mutagenesis, transposons can generate non-coding RNAs (e.g., miRNAs) and functional proteins (e.g., transposase, reverse transcriptase, and envelope proteins) that further destabilize the genome and perturb cellular homeostasis, contributing to cancer and other diseases.

Transposons can provide enhancer and promoter elements that regulate host gene expression, alter 3D chromatin structure, or generate new regulatory elements, including non-coding RNAs and microRNAs (Fueyo et al., 2022).One of the primary mechanisms through which transposons can contribute to carcinogenesis is by activating oncogenes (Jang et al., 2019). Oncogenes are genes that, when mutated or overexpressed, can drive abnormal cell growth and proliferation (Shortt and Johnstone, 2012). Transposons can insert near these oncogenes, disrupting their regulatory sequences and leading to their aberrant activation (Rodriguez-Martin et al., 2020) (**Figure 1a**). These dysregulations can result in the continuous stimulation of cell growth signals, promoting uncontrolled division of cells, a hallmark of cancer (Lee et al., 2024). It has been shown that long terminal repeat sequences (LTRs) of Human endogenous retroviruses (HERVs) can act as alternative promoters or enhancers to drive the expression of neighboring genes, including those associated with tumorigenesis (Zhang et al., 2019). In Hodgkin’s lymphoma, an aberrant activation of the “mammalian apparent LTR retrotransposon”(MaLR) family transposon-like human element 1B (THE1B) due to the disruption of epigenetic control by deletion of co-blocker protein CBFA2T3, promotes transcription of the non-B, myeloid-specific proto-oncogene colony-stimulating factor 1 receptor (CSF1R). CSF1R expression is critical for the survival of B-cell- derived Hodgkin’s lymphoma cells (Lamprecht et al., 2010).

Transposons have important roles in the transcriptional regulation of human genes and may promote carcinogenesis. In triple-negative breast cancer (TNBC), certain transposon subfamilies provide abundant binding sites for oncogenic transcription factors, accompanied by active histone modifications such as H3K27ac that maintain open chromatin states for transcription. This interplay likely facilitates oncogene activation and contributes to genomic instability, promoting cancer progression. These findings align with the concept that transposons disrupt genomic stability and may drive tumorigenesis in TNBC by facilitating sustained oncogene expression (Huang et al., 2021). Moreover, individual **t**ransposons may have promoter activity. For example, **t**ransposons located upstream of *SYT1*, *UCA1*, *AK4* and *PSAT1* were identified with promoter activity in three TNBC cell lines (MDA-MB-468, MDA-MB-231 and BT549). L1PA2 (a kind of transposon), when hypomethylated, controls *SYT1* expression, while deletion of L1PA2 results in an almost complete loss of *SYT1* in MDA-MB-468 and MDA-MB-231 cells (Jiang and Upton, 2019). Primate-specific L1 transposons play a role in transcriptional regulation of carcinogenesis. In MCF7 breast cancer cell line, L1 transposons recruit a variety of cancer-specific transcription factors, primarily binding to their 5′UTR (Jiang et al., 2021), thus it is possible that L1 transposon serve as hosts for transcription factors to oncogene and drive tumorigenesis.

Some transposons act as cryptic promoters that are reactivated by epigenetic mechanisms (e.g. DNA methylation) and thus drive oncogene expression in cancer, a process known as onco-exaptation. It has been shown that the AluJb transposon acts as a switch for AluJb-P activity and drives *LIN28B* expression as an alternative promoter through dynamic DNA methylation regulation in lung cancer cell lines (Jang et al., 2019). Furthermore, onco-exaptation events involving transposons are widespread across various cancer types and are closely associated with tumorigenesis. For example, in Hodgkin lymphoma (HL), upregulation of the transcription factor *interferon regulatory factor 5* (*IRF5*) is driven by transcriptional activation induced by an altered epigenetic state of open and hypomethylated endogenous retroviral LOR1a long terminal repeat (LTR) that are normally dormant upstream of *IRF5 (*Babaian et al., 2016).

Many cell signaling pathways are critical for regulating essential cellular processes such as cell growth, proliferation, differentiation, and apoptosis (Su et al., 2024). Dysregulation of these pathways can contribute to cancer by promoting uncontrolled cell division and inhibiting cell apoptosis (Sever and Brugge, 2015). Transposons can affect cell signaling pathways that may be implicated in cancer development (Anwar et al., 2017). Transposons have a potential to disrupt normal mediator expressions of cellular signaling by inserting themselves into or near the genes involved in these pathways, altering their expression or function (Du et al., 2023). Due to transposon insertions, the accumulation of these genetic alterations can disrupt key signaling pathways involved in cell growth, differentiation, and apoptosis, providing cancer cells with a selective advantage and promoting their clonal expansion (Grundy et al., 2022). For example, transposon-mediated activation of oncogenes such as *ras* or *myc* can hyperactivate genes of signaling molecules like the MAPK/ERK pathway or the PI3K/AKT/mTOR pathway, of which activities are commonly dysregulated in various cancers (Scott and Devine, 2017). Additionally, a retrotransposon Alu element can regulate gene expression by providing a classical polyadenylation signal (Beaudoing et al., 2000; Lopez et al., 2006). In addition, the structural variation caused by its insertion leads to the development of a wide range of complex diseases, including cancer (Larsen et al., 2017; Payer et al., 2017).

The frequency of chromosomal translocations is significantly higher when active L1 is present in the cell. These translocations preferentially occur at transcription end sites of active genes, early replication regions, and accessible chromatin regions (Tao et al., 2024). Emerging evidence suggests that transposons may also modulate the tumor microenvironment, influencing immune responses and promoting tumor progression (Schauer et al., 2018). Mechanistically, they alter the expression of immune-related genes, leading to secretion and release of pro-inflammatory cytokines (Alkailani and Gibbings, 2023).

Studies have shown that hypomethylation of L1 elements in cancer cells can lead to increased transposon activity and contribute to the development and progression of various cancers (Baba et al., 2024). For example, a recent study demonstrated that hypomethylation of a specific L1 promoter induces an alternate transcript of the *MET* oncogene in bladder tumors and urothelial tissues of tumor-bearing bladders, promoting carcinogenesis (Wolff et al., 2010). Additionally, research has shown that hypomethylation of L1 elements in ovarian cancer contributes to increased retro-transposition events and genomic instability (Rodić et al., 2015). Similarly, L1 hypomethylation is associated with poor prognosis in esophageal squamous cell carcinoma (ESCC) and gastric cancer (Iwagami et al., 2013; Shigaki et al., 2013). All these studies indicate that L1 hypomethylation/high activity may play a critical role in promoting tumorigenesis.

## 5 Transposons also dysregulate cellular processes by repressing gene expression

Conversely, transposons can also disrupt tumor suppressor genes, which act as guardians of the genome by inhibiting cell cycle progression and promoting DNA repair and apoptosis (Aiderus et al., 2021) (**Figure 1b**). The loss of genomic stability can further drive tumorigenesis by creating a mutational landscape conducive to cancer development. For example, a novel somatic L1 insertion at the *APC* gene in colorectal cancer (CRC) may play a potential role for retrotransposons in tumor initiation through a two-hit pathway. The study also highlights population-specific variations in L1 source profiles, indicating a new aspect of cancer risk (Scott et al., 2016). It is notable that L1 plays a suppressive role in tumorigenesis of acute myeloid leukemia (AML). A study indicated that MPHOSPH8/MPP8, as a component of human silencing hub (HUSH) complex, silences L1 by epigenetically modifying its promoter. Therefore, silencing L1 repressed development of myeloid leukemia (Gu et al., 2021). In upper gastrointestinal cancers, L1 hypomethylation not only increases the reverse transcription of L1 RNA into DNA, leading to its reintegration into the genome, but also results in an increase in tumor-specific insertions. These insertions commonly occur in genomic regions associated with tumor suppressor genes, thus promoting cancer development by suppressing the function of these anti-oncogenes (Baba et al., 2024).

It has been shown that some transposons cause epigenetic reprogramming of neighboring gene promoters(**Figure 1c**). The retrotransposon SINE B1 element promotes transcriptional repression of genes in close proximity to their promoters. At this point, DNA methylation occurs in the promoter region, but this is not due to methylation spreading outwards from the SINE B1 sequence, but is mediated by other mechanisms. This regulation may have adverse effects on the cell. At the same time, the presence of SINE B1 in the vicinity of a gene promoter is usually subject to negative selection unless the promoter region is protected by an insulator element, such as a binding protein like CTCF (Estécio et al., 2012).

Additionally, transposon insertions can disrupt the gene sequences encoding key components involved in signaling pathways in cancer, such as the Wnt/β-catenin pathway, Notch pathway, Hedgehog pathway, and TGF-β signaling pathway (Xia et al., 2022). Alterations in these pathways can promote tumorigenesis by driving abnormal cell behaviors and disrupting tissue homeostasis (You et al., 2023).Transposon insertions near genes encoding components of these pathways can lead to their abnormal activation or inactivation, contributing to oncogenic transformation (Ranzani et al., 2013). Transposon-induced disruption of tumor suppressor genes like *TP53* or *PTEN* can impair their activity to inhibit these signaling pathways, leading to sustained activation of these pathways (Hancks and Kazazian, 2016). The long non-coding RNA MER52A derived from the MER52A retrotransposons is specifically overexpressed in hepatocellular carcinoma (HCC). lncMER52A affects the expression of genes related to the epithelial-mesenchymal transition (EMT) signaling pathway, and is able to bind to p120-catenin (also known as CTNND1) to activate the p120-ctn/Rac1/Cdc42 axis and promote invasion and metastasis of HCC cells (Wu et al., 2020).

## 6 RNAs or peptides derived from transposons may interfere with normal cellular functions

Proteins/peptides encoded by transposons affect cellular biological functions through a variety of mechanisms(**Figure 1d**), e.g., interfering with gene expression, participating in DNA damage/repair, epigenetic alterations, activating cellular signaling pathways, regulating the cell cycle, and triggering immune responses. TnpB and IscB proteins encoded by transposon act as a nuclease that helps itself propagation, directly play some roles or interact with other proteins and may interfere with other protein function in cells (Meers et al., 2023). An LTR transcript in diffuse large B-cell lymphoma (DLBCL), encoding fatty acid binding protein 7 (FABP7), promotes cancer development (Lock et al., 2014). Reactivation of the ancient LTR was found to promote a novel oncogenic transcript of *ERBB4* in ALK-negative anaplastic large cell lymphoma (ALCL). Aberrant *ERBB4* transcripts in a subset of ALK-negative anaplastic large cell lymphoma (ALCL) are driven by reactivated intronic long terminal repeats (LTRs), highlighting how endogenous transposons can disrupt transcriptional regulation, destabilize the genome, and contribute to oncogenic transformation (Scarfò et al., 2016). Furthermore, the transposon-encoded protein TnpB not only catalyzes the cleavage and rejoining of DNA, but also acts as a ribonuclease, processing its own mRNA to produce a mature non-coding RNA, known as omega RNA. Omega RNA binds to TnpB to form a ribonucleoprotein complex, which can recognize and cleaving specific DNA sequences (Nety et al., 2023).

Moreover, HERVs contribute to the development of diseases, including cancer, by encoding proteins that act as promoters/enhancers or lncRNAs. HERV envelope proteins can induce intercellular fusion or epithelial mesenchymal transition, promoting tumorigenesis and progression in melanoma, endometrial and breast cancer (Grandi and Tramontano, 2018). It has been identified that several HERV-derived lncRNAs, such as UCA1, SAMSON, and BANCR, are involved in bladder and melanoma carcinogenesis (Mao et al., 2021).In addition, in cells infected with SARS-CoV-2, exons from transposons produce the immune-gene functional protein variant of the type I interferon receptor IFNAR2 that effectively inhibit interferon signaling activity (Pasquesi et al., 2023).

Transposons, particularly L1 elements, have been implicated in various aspects of carcinogenesis (Chenais, 2015). Transcriptional activation of the L1 element itself can alter other gene expression, activate oncogenes, disrupt tumor suppressor genes, and contribute to genomic instability, all of which can promote tumorigenesis (Xiao-Jie et al., 2016). In addition, the proteins or peptides encoded by these elements may disrupt cellular biological functions, potentially leading to tumorigenesis (Liao et al., 2023). For example, L1 transposon ORF2 encodes a protein with endonuclease (EN) and reverse transcriptase (RT) activity that cleaves genomic DNA, forming double-strand breaks (DSBs). These breaks usually occur at or near the L1 insertion site, and DSBs are a major source of genomic instability. Such breaks trigger the DNA damage response (DDR); however, if the repair process fails or is erroneous, unrepaired breaks can lead to changes in genome structure, including chromosomal rearrangements, deletions, or mutations (Hedges and Deininger, 2007; Mendez-Dorantes et al., 2024).

Non-coding RNAs derived from transposons, such as long non-coding RNAs(LncRNAs), are involved in key cellular signaling pathways. These RNAs interact with various transcription factors, such as *p53*, *Sp1*, and *NF-Y*, to modulate cell cycle processing, contributing to cell proliferation and potentially influencing cell cycle arrest mechanisms (Xu et al., 2013). For example, Alu RNA is able to bind to RNA polymerase II under cellular stress and regulates expression of stress-responsive genes (Ponicsan et al., 2010).

## 7 Targeting transposons represents a potential therapeutic approach in cancer

Transposons have become key players in cancer progression. Given their central role in tumorigenesis, transposon-targeted therapies, particularly those utilizing reverse transcriptase inhibitors and epigenetic regulators, represent a novel avenue in cancer treatment (Wang et al., 2024). In colorectal cancer, retro-transposition events occur frequently and may be closely associated with tumorigenesis. Studies have demonstrated that the nucleoside reverse transcriptase inhibitor (NRTI) lamivudine (3TC) targets L1 retrotransposons in preclinical models of colorectal cancer, inhibiting their reverse transcriptase activity, and thereby helping to maintain genomic stability (Rajurkar et al., 2022). However, when tumors have undergone immune evasion and immune recognition needs to be reactivated, epigenetic drugs such as DNA demethylating agents or histone deacetylase inhibitors can be employed to reactivate silenced transposons and induce their expression. Jang et al. demonstrated that epigenetic drugs can enhance the immunogenicity of glioblastoma cells by reactivating silenced transposable elements, leading to the production of therapy-induced transposon-derived antigens (TI-TEAs), which may synergize with existing immunotherapeutic approaches (Jang et al., 2024).

ERV sequences retain open reading frames encoding full-length retroviral proteins. Epigenetic dysregulation in cancer induces abnormal ERV expression across multiple tumour types. Consequently, ERVs possess immunogenicity and high expression in cancer cells (compared to normal tissue), making them promising therapeutic targets in cancer treatment (Gracia-Hernandez et al., 2025). Wang-Johanning and colleagues developed a monoclonal antibody targeting the HERV-K Env protein, which is expressed in breast cancer cell lines and patient samples. Their research demonstrated that this antibody effectively inhibited breast cancer cell proliferation and induced apoptosis *in vitro*, and also significantly suppressed tumor growth in *in vivo* xenograft models (Wang-Johanning et al., 2012). In preclinical models, Krishnamurthy and colleagues engineered T cells to express a chimeric antigen receptor (CAR) derived from a monoclonal antibody targeting HERV-K Env. They discovered that these specific CAR-T cells were capable of reducing tumor growth in HERV-K Env+ metastatic melanoma models (Krishnamurthy et al., 2015).

Somatic transposon insertions may promote the development of various cancers by upregulating oncogenes or inducing genomic rearrangements. Analysis of recently mobilized transposon subfamilies (RMS) can be used to screen for potentially pathogenic insertions, providing a theoretical basis for exploring novel cancer therapeutic strategies. For example, it is conceivable to use CRISPRi to downregulate oncogenic transcripts driven by transposons promoters resulting from somatic insertions (Autio et al., 2021). In addition, it is important to note that when CRISPR/Cas9 is used for targeted gene therapy, its induction of double-strand breaks (DSBs) at the editing site may frequently trigger insertion events of the L1 retrotransposon if active L1 reverse transcriptase (RT) is present in the cell. Such insertions could contribute to genomic instability, posing a potential safety risk in CRISPR/Cas9 applications (Tao et al., 2022).

Moreover, it is noticeable that transposons can facilitate the horizontal transfer of oncogenic sequences between cells, contributing to the genetic heterogeneity observed within tumors (Evrony et al., 2015). This genetic diversity can confer resistance to therapeutic interventions, as different cell populations within the tumor may harbor distinct oncogenic mutations, making them less susceptible to targeted treatments.

## 8 Perspective

Transposons are no longer dismissed as mere “junk DNA”; instead, they are increasingly recognized as dynamic genomic players with profound implications for disease. While their role in disrupting genomic stability is well-documented, emerging research suggests that transposons may also contribute to disease through more nuanced mechanisms, including immune activation, epigenetic reprogramming, and intercellular transfer. Future investigations should explore these understudied aspects to help uncover novel therapeutic strategies.

Recent studies reveal that transposon-derived nucleic acids can activate innate immune sensors (e.g., TLRs, cGAS-STING), triggering chronic inflammation—a hallmark of aging, cancer, and autoimmune diseases. Could transposon suppression mitigate inflammatory conditions like rheumatoid arthritis or lupus? Further research into transposon-immune crosstalk may identify anti-inflammatory therapies targeting retroelement silencing.

Somatic transposon mobilization in neurons has been linked to neurodegenerative diseases, but its extent and consequences remain unclear. Single-cell sequencing and CRISPR-based lineage tracing could map transposon activity in aging brains, clarifying whether L1 retro-transposition accelerates cognitive decline. If so, reverse transcriptase inhibitors (e.g., those used for HIV) might be repurposed to protect neuronal genomes. Beyond traditional insertional mutagenesis, transposons may spread between cells via extracellular vesicles, facilitating cancer heterogeneity and therapy resistance. Investigating horizontal transposon transfer could reveal new mechanisms of tumor evolution and biomarkers for metastasis.

Since DNA hypomethylation activates transposons, epigenetic drugs (e.g., DNMT inhibitors) may have paradoxical effects—both suppressing and reactivating transposons. Developing selective transposon silencers, such as small RNAs or CRISPR-dCas9-based epigenetic editors, could offer precision control over pathogenic elements without disrupting beneficial genomic regulation. The complexity of transposons-mediated diseases necessitates interdisciplinary approaches, combining genomics, immunology, and computational biology. By deciphering how transposons shape disease trajectories, we may unlock innovative treatments that restore genomic stability and halt pathological inflammation. Future research must prioritize translating these insights into clinical interventions, potentially revolutionizing therapy for cancer, neurodegeneration, and autoimmune disorders.
